# Supplementary material for: Mechanisms governing the pioneering and redistribution capabilities of the non-classical pioneer PU.1
Source: Nat Commun. 2020 Jan 21;11:402. doi: 10.1038/s41467-019-13960-2 (PMC6972792; doi:10.1038/s41467-019-13960-2)
Supplement: Supplementary file 7 — Source data [file 41467_2019_13960_MOESM7_ESM.zip › Source_Data/FigureS2/FigureS2B_MotifScanOutput/KmeansCluster10/homerResults/motif3.similar.html]

motif3

## Information for motif3

T
C
G
A
T
C
G
A
T
C
G
A
C
G
T
A
T
C
A
G
A
G
T
C
C
T
G
A
C
T
A
G
G
C
T
A
G
C
T
A
T
C
A
G
C
A
G
T
T
A
C
G
  
Reverse Opposite:  

A
T
G
C
G
T
C
A
A
G
T
C
C
G
A
T
C
G
A
T
A
G
T
C
A
G
C
T
T
C
A
G
A
G
T
C
G
C
A
T
A
G
C
T
A
G
C
T
A
G
C
T
  

|  |  |
| --- | --- |
| p-value: | 1e-130 |
| log p-value: | -3.003e+02 |
| Information Content per bp: | 1.708 |
| Number of Target Sequences with motif | 351.0 |
| Percentage of Target Sequences with motif | 9.46% |
| Number of Background Sequences with motif | 698.0 |
| Percentage of Background Sequences with motif | 1.58% |
| Average Position of motif in Targets | 99.8 +/- 37.4bp |
| Average Position of motif in Background | 100.3 +/- 69.4bp |
| Strand Bias (log2 ratio + to - strand density) | -0.2 |
| Multiplicity (# of sites on avg that occur together) | 1.01 |
| Motif File: | file (matrix) reverse opposite |

### Similar de novo motifs found

|  |  |  |  |  |  |  |  |
| --- | --- | --- | --- | --- | --- | --- | --- |
| Rank | Match Score | Redundant Motif | P-value | log P-value | % of Targets | % of Background | Motif file |
| 1 | 0.869 | C G T A A T G C C G A T C G A T A G T C A G C T T A C G G A T C G C A T G C A T G C A T | 1e-81 | -187.915619 | 11.18% | 3.50% | motif file (matrix) |
| 2 | 0.933 | A T G C G T C A A T G C C G A T A C G T A G T C A G C T A T G C A G T C G C A T A C G T A G C T A G C T A G C T | 1e-53 | -123.462506 | 2.94% | 0.32% | motif file (matrix) |
| 3 | 0.821 | G A C T T C G A C T A G C T G A C G T A T C G A A G T C A C T G C T A G C G T A C G T A C T A G A C G T A C T G | 1e-25 | -58.448548 | 1.02% | 0.06% | motif file (matrix) |
